# Emotional modulation of experimental pain: a source imaging study of laser evoked potentials

**DOI:** 10.3389/fnhum.2013.00552

**Published:** 2013-09-17

**Authors:** Andrej Stancak, Nicholas Fallon

**Affiliations:** Department of Experimental Psychology, Institute of Psychology, Health, and Society, University of LiverpoolLiverpool, UK

**Keywords:** EEG, cortex, source dipole analysis

## Abstract

Negative emotions have been shown to augment experimental pain. As induced emotions alter brain activity, it is not clear whether pain augmentation during noxious stimulation would be related to neural activation existing prior to onset of a noxious stimulus or alternatively, whether emotional stimuli would only alter neural activity during the period of nociceptive processing. We analyzed the spatio-temporal patterns of laser evoked potentials (LEPs) occurring prior to and during the period of cortical processing of noxious laser stimuli during passive viewing of negative, positive, or neutral emotional pictures. Independent component analysis (ICA) was applied to series of source activation volumes, reconstructed using local autoregressive average model (LAURA). Pain was the strongest when laser stimuli were associated with negative emotional pictures. Prior to laser stimulus and during the first 100 ms after onset of laser stimulus, activations were seen in the left and right medial temporal cortex, cerebellum, posterior cingulate, and rostral cingulate/prefrontal cortex. In all these regions, positive or neutral pictures showed stronger activations than negative pictures. During laser stimulation, activations in the right and left anterior insula, temporal cortex and right anterior and posterior parietal cortex were stronger during negative than neutral or positive emotional pictures. Results suggest that negative emotional stimuli increase activation in the left and right anterior insula and temporal cortex, and right posterior and anterior parietal cortex only during the period of nociceptive processing. The role of background brain activation in emotional modulation of pain appears to be only permissive, and consisting in attenuation of activation in structures maintaining the resting state of the brain.

## Introduction

Previous studies showed pain augmentation during negative emotions induced by unpleasant odors (Villemure et al., 2003), depressing verbal statements (Zelman et al., 1991; Berna et al., 2010), unpleasant sounds (Stancak et al., 2013), depressed music (Tang et al., 2008), or negative visual scenes (de Wied and Verbaten, 2001; Kenntner-Mabiala and Pauli, 2005; Godinho et al., 2006; de Tomasso et al., 2008; Kenntner-Mabiala et al., 2008; Roy et al., 2009; Ploner et al., 2011). However, current understanding of neural mechanisms of pain modulation by induced emotions is far from complete. Functional MRI and PET studies [reviewed in Wiech and Tracey (2009)] pointed to a number of brain regions mediating emotional modulation of pain, including amygdala (Roy et al., 2009; Berna et al., 2010), hippocampal formation (Ploghaus et al., 2001; Moulton et al., 2011), cerebellum (Moulton et al., 2011), and anterior insula (Roy et al., 2009; Moulton et al., 2011; Ploner et al., 2011). Recently, functional networks such as cerebellum-anterior insula-hippocampal formation (Moulton et al., 2011) or anterior insula-medial temporal cortex (Ploner et al., 2011) have been postulated to account for modulation of pain in emotional context.

Two alternative neurophysiological models of emotional modulation of pain can be postulated. The *background activation model* would predict that select brain regions participating in processing and maintenance of emotional states would increase the baseline activation in pain processing networks of the brain in a valence-dependent manner. The background activation model would postulate that regions, known to participate in pain processing, such as anterior insula or anterior cingulate cortex, would show an elevated activation already prior to arrival of a noxious stimulus. Recent reports on the role of pre-stimulus functional connectivity between anterior insula and brain stem (Ploner et al., 2010) and low amplitude of pre-stimulus amplitude of alpha band EEG oscillations (Babiloni et al., 2003) in predicting laser-induced pain would accord with the background activation model. Alternatively, *nociceptive processing model* would assume that emotional modulation of pain takes place only at the stage of cerebral processing of a noxious stimulus. In this model, negative emotional states would increase the gain in critical brain regions participating in pain processing, causing an augmented neural output upon arrival of a nociceptive stimulus.

The two suggested models of emotional modulation of pain derive primarily from temporal segregation of brain activations relative to onset asynchronies of emotional and nociceptive stimuli. In the framework of the recent conceptual act theory of emotion (Barrett, 2006, 2009; Barrett and Bliss-Moreau, 2009), the background activation model would refer to core affect underlying continuous conscious experience. In contrast, the nociceptive processing model of pain modulation, in the framework of conceptual act theory, would refer to the period of fast conceptualization process initiated by the arrival of a noxious stimulus. This conceptualization encompasses features of both the nociceptive stimulus and the context, and leads to categorization of a somatosensory sensation as pain. As a noxious laser stimulus is an aversive stimulus, we hypothesize that a negative emotional picture compared to a neutral or positive picture would shift the valence of core affect toward the negative pole, and this comparatively negative core affect would in turn allow a direct and fluent categorization of the noxious stimulus as pain. Thus, the high compatibility between core affect and pain conceptualization predicts greater pain when a noxious stimulus occurs on the background of a negative emotion compared to a situation when a noxious stimulation arrives on the background of a positive or neutral emotional stimulus. This hypothesis is also in line with both experimental studies showing greater amplitude of defensive acoustic startle reflex during viewing negative emotional pictures (Bradley et al., 2006; Löw et al., 2008), and the motivation-action theory of emotion (Lang et al., 1998) postulating that emotions entail lines of motivated behavior, such as approach or avoidance, which prepare the organisms for appropriate action. Enhancement of pain in the presence of negative emotions may help to boost avoidance behavior, potentially facilitating termination of, or withdrawal from, a noxious stimulus.

To evaluate the validity of each model, information about temporal aspects of nociceptive processing and effects of emotional stimuli at different stages of nociceptive processing is of importance. However, the limited time resolution of fMRI, equivalent to the scale of seconds (Kim et al., 1997), does not allow differentiation of cerebral activation changes occurring on a scale of tens or few hundreds of milliseconds, which processes underlie emotional modulation of a brief noxious stimulus.

To analyse effects of emotional stimuli on brain activation changes occurring prior to and during nociceptive processing, we decided to analyse averaged EEG potentials in the peri-stimulus interval just preceding arrival of a noxious stimulus, and during the period of central processing of a noxious laser stimulus. To resolve region-specific time courses of activation in large clusters of activation, we employed a combination of distributed source dipole modeling and independent component analysis methods. In contrast to source multiple source dipole modeling methods based on sets of equivalent source dipoles (Scherg and Von Cramon, 1986), distributed source models [reviewed in Michel et al. (2004) and Grech et al. (2008)] evaluate thousands of dipoles having fixed locations in a grid of voxels, and this method does not require any subjective decisions about the plausibility of individual source dipole models. It was envisaged that a large number of sources would operate in our data due to concurrent effects of visual and somatosensory stimuli, emotions, and stimulus anticipation. Therefore, in the present study we decided to employ distributed source dipole analysis. Distributed source dipole models are computed in select time intervals based on the latency of a defined event-related potential component or obtained using a clustering algorithm (Murray et al., 2008). In the present study, we chose to explore full evoked potential data by first computing source image maps in every time point of LEP and subsequently identifying spatial locations and temporal activation profiles of a number of statistically independent clusters using ICA. Our method bears some similarity with a previous study employing ICA to scalp EEG potentials and subsequently localizing the sources of independent components using distributed source dipole modeling (Gomez-Herrero et al., 2008). In contrast to previous studies (Supp et al., 2007; Gomez-Herrero et al., 2008), cluster separation was performed with source image data obtained over a comparatively long time interval to allow ICA to utilize information about specific, statistically independent components potentially existing in larger activation clusters.

We hypothesized that if activation components operating prior to laser stimulus would account for emotional modulation of pain, their strength would vary in parallel with emotional type of pictures both prior and after stimulus onset. Alternatively, if emotional modulation would take place during the period of cerebral nociceptive processing, only activation sources seen during LEP period would change in parallel with emotional valence of pictures.

## Methods and materials

### Subjects and procedure

Sixteen healthy subjects (7 females, 9 males, mean age 23.9 ± 2.7 years, mean ± *SD*) with normal vision took part in the study after giving their informed consent in accordance with the Declaration of Helsinki. The study was approved by the Research Ethics Committee of the University of Liverpool. All but two subjects had right-hand dominance according to self-report.

Subjects were seated in a sound and light attenuated room and viewed a 19 inch computer monitor placed 0.7 m in front of them. The subject's right hand rested on a wooden desk and a circle of 3 cm in diameter was drawn on the dorsolateral part of the hand. One experimenter sat next to the subject but out of view. The experimenter held the hand piece of the laser stimulator and orientated the laser beam by changing pseudo-randomly the target spot within the defined area on the hand.

Forty negative, positive, and neutral pictures were selected from the International Affective Picture System (http://csea.phhp.ufl.edu/media/iadsmessage.html) containing samples of validated affective pictures. To avoid any association between pain stimuli and somatosensory stimulation, we have not used pictures showing disfigured bodies, blood, wounds, or erotic scenes. Avoiding pictures with erotic content lead to slightly smaller arousal ratings in positive compared to negative pictures. To account for potential independent effects of picture valence and arousal, subjects evaluated all pictures for valence and arousal using a 9-point Self-Assessment Manikin scale (Lang et al., 2008) after the experiment. The arousal and valence ratings were used together with pain ratings as covariates during statistical analysis whenever an effect of pictures in one-way ANOVAs reached statistical significance.

The experiment was organized into 4 blocks each lasting 7 min. In one block, 30 trials of 13 s durations were presented. Each trial began with a 4-s resting period during which subjects viewed a black fixation cross on a light gray background. This was followed by a 3-s picture viewing period. A noxious laser stimulus was applied to the dorsum of the right hand at 1100 ms following onset of a photograph. This laser stimulus onset time was selected because the picture-induced event-related potentials show only a sustained and stable “late positive potential” at this stage with earlier evoked potential components terminating <600 ms following presentation of pictures (Foti et al., 2009). A constant interval between onset of a picture and subsequent laser stimulus implied the presence of pain anticipation. Since this interval was identical in every emotional condition, we assumed that the effects of pain anticipation itself would not account for any differences in pain or cortical responses among the three types of emotional pictures. Each picture stimulus was followed by a brief resting period (2 s) and an evaluation period of 4 s during which subjects evaluated the pain perceived during the last trial. A seven-point rating scale with anchors “no pain at all” (1) and “worst possible pain” (7) was presented in the form of seven horizontally aligned dark gray rectangles appearing on light gray background. Subjects rated the intensity of their pain by repeatedly pressing a computer mouse with their left hand to increment the scale.

Laser stimuli were applied using Nd-YAP laser stimulator (Stim1324, El.En., Italy). The pulse duration was 2 ms, and the spot size was 4 mm. The intensity of laser stimulus was adjusted individually prior to the first block by incrementing the stimulus intensity from 1.25 to 2.0 J in 0.25 J steps. The intensity producing a moderate pain sensation, rated 3-4 on a 7-point rating scale, was used throughout. These stimulus parameters were optimized to produce a sharp pricking pain mediated by Aδ fibers.

After the experiment, subjects rated valence and arousal associated with each picture using 9-point Self-Assessment Manikin scales (Lang et al., 2008). The Pain Catastrophising Scale (Sullivan et al., 1995) and the State and Trait Anxiety Inventory (Spielberger et al., 1970) were administered to establish that our sample fell within the ranges of the normal population in measures potentially affecting pain and emotions.

### Recordings

EEG was recorded continuously using the 128-channel Geodesics EGI System (Electrical Geodesics, Inc., Eugene, Oregon, USA) with the sponge-based Geodesic Sensor Net. The sensor net was aligned with respect to three anatomical landmarks including two pre-auricular points and the nasion. The electrode-to-skin impedances were kept below 50 kΩ and at equal levels in all electrodes. The recording bandpass filter was 0.1-200 Hz, and the sampling rate was 1000 Hz. The electrode Cz was used as the reference.

EEG data were processed using BESA v. 5.3 program (MEGIS GmbH, Munich, Germany). Data were first transformed off-line into reference-free data using common average method. The oculographic and, when necessary, electrocardiographic artifacts were removed by principal component analysis (Berg and Scherg, 1994) based on averaged eye-blink and ECG artifact topographies obtained using a template matching algorithm. Data were visually inspected for presence of any movement or muscle artifacts, and epochs contaminated with artifacts were excluded.

To analyse the period of nociceptive processing, we employed epoch durations and filters suitable for analysis of LEPs. Thus, LEPs were computed separately for each of three emotional conditions by averaging the respective epochs in the intervals ranging from 300 ms before stimulus onset to 800 ms after stimulus onset (1100 samples). The baseline period ranged from −300 to 0 ms relative to the onset of laser stimulus. Evoked potentials from four blocks were averaged and LEPs were filtered from 2 to 35 Hz. The mean number of epochs used for averaging was 32.2 ± 5.9 (mean ± *SD)* during neutral, 31.7 ± 5.4 during negative, and 31.3 ± 5.3 during positive emotional picture conditions.

To evaluate event-related potential preceding and co-occurring with onset of laser stimulus, another set of epochs was created encompassing 800 ms before and 700 ms after stimulus onset (1500 samples). Data were bandpass filtered from 0.1 to 30 Hz and averaged using the interval from −800 to −500 ms as a baseline. This baseline period was chosen after checking in preliminary analyses that the interval was stable and devoid of short- and mid-latency picture-induced evoked potential components. The long data epoch allowed us to analyse brain activations occurring during an interval affected by emotional pictures and pain anticipation, and to identify components that were active during the LEP period and prior to laser stimulation.

### Source dipole reconstruction

In both types of data, LEPs and the peri-stimulus potentials, sources of EEG potentials were evaluated at every time point using local autoregressive average method (Grave De Peralta Menendez et al., 2001, 2004; Grave De Peralta Menendez and Gonzalez Andino, 2002). In LAURA, vector fields at each point of the brain are computed, based on physical laws governing potentials and currents in a biomedical medium, by applying a local autoregressive operator with a decay corresponding to the power of the distance (Grave De Peralta Menendez and Gonzalez Andino, 2002). LAURA yields a large number of sources with zero dipole localization errors and outperforms other localization algorithms such as the minimum-norm or the Laplacian-operator method (Grave De Peralta Menendez et al., 2001; Grave De Peralta Menendez and Gonzalez Andino, 2002). In a preliminary analysis, the LAURA activation maps of major LEP components were compared with the standardized low-resolution tomography analysis method (Pascual-Marqui, 2002). Both methods yielded identical maxima for the main clusters, however, in our particular type of data LAURA resolved additional spatial clusters and therefore, this method was preferred.

LAURA was evaluated using BESA v. 5.3. program in a grid of 4452 voxels sized 7 × 7 × 7 mm^3^ covering the whole brain including cerebellum. Several regularization constants in the range of 0–1% were tested in pilot computations. The default value of regularization of 0.03% was chosen as the one yielding solid, yet separate clusters. Volumes series counting 700 volumes (from −100 to 600 ms; bandpass filter 2–35 Hz) were computed in every subject and in each of three emotional conditions to analyse activation changes underlying LEPs. Trimming the LEP epoch allowed us to reduce the volume of data entering ICA, and to focus on data points containing strong potential components. Another set of 1500 LAURA volumes (from −800 to 700 ms; bandpass filter 0.1–30 Hz) was created to evaluate the activation changes occurring prior to and at onset of laser stimulus.

### Independent component analysis

Volumes of LAURA maps in the time epoch from −100 to 600 ms (700 volumes), computed in 16 subjects and 3 conditions, were analyzed using group ICA (Calhoun et al., 2001). In group ICA, the full data was first reduced by principal component analysis to 30 principal components which have been subsequently analyzed using Infomax algorithm in GIFT program (http://icatb.sourceforge.net). To estimate the number of ICs, ICASSO algorithm (Himberg et al., 2004) with 15 ICA repetitions was run. Eventually, the number of ICs was set to 14 based on internal consistency of independent components. However, pilot computations with a range of component numbers (3, 6, 8, 10, 12, 20) were also run to check that the number of ICs, decided according to ICASSO results, was optimal. The component *T*-maps were mildly smoothed and converted to *Z*-maps and visualized in GIFT program with the threshold of *Z* = 3.1. To assign anatomical labels to ICs cluster maxima, individual component maps obtained in each of 16 subjects were also analyzed using an univariate *T*-test in SPM8 (The Welcome Trust for Neuroimaging, Institute of Neurology at UCL; http://www.fil.ion.ucl.ac.uk). The *T*-maps were thresholded rigorously with *T* = 70, roughly corresponding to a family-wise error correction of *P* = 10^−6^. The cluster coordinates, identified in SPM maps, were assigned anatomical labels using TalairachDaemon (University of Texas Health Science Center; www.talairach.org/daemon.html) and Anatomy toolbox (Forschungszentrum Jülich, Germany; www.fz-juelich.de/inm/inm-1/spm_anatomy_toolbox). ICs time courses in each subject and emotional condition were saved for further statistical analysis.

To analyse the potential changes occurring in the peri-stimulus interval and during the first 100 ms after onset of laser stimulus, LAURA volumes were also computed in epochs ranging from −800 to 700 ms. This long epoch was chosen to analyse whether brain regions showing stronger activation during negative than positive or neutral pictures prior to onset of laser stimulus would also show similar enhancements during the period of LEP. These epochs were filtered from 0.1 to 30 Hz, and averaged using the period from −800 to −500 ms as a baseline. Computation of group ICA was similar to LEP period data. Initially, 20 ICs were computed and after running ICASSO algorithm, the number of ICs was set to 17 based on internal consistency of individual ICs over 15 ICA runs. The statistical threshold in univariate group *T*-test was set to 50.

### Statistical analysis

To evaluate effects of emotional pictures on behavioral measures and source activations, a one-way ANOVA for repeated measures was used. All *P*-values from ANOVA analyses were adjusted with Greenhouse–Geisser e correction to account for violation of the assumption of sphericity. Student's paired *t*-test was used to compute the contrasts between two emotional picture conditions. To analyse whether a change in source activation components would be associated with subjective reports of pain, picture valence, and arousal, one-way repeated measures analysis of covariance was computed in BMDP2V program (Statistical Solutions Ltd., Cork, Ireland). This analysis used the amplitude of a particular independent component as a dependent measure and pain and picture valence and arousal as covariates. The covariance analysis also accounted for potential inter-correlations between covariates.

The IC waveforms were analyzed using one-way repeated measures ANOVA at each time point in the range from −100 to 600 ms in LEP period data and from −500 to 100 ms in peri-stimulus period data. The statistical significance was evaluated using permutation method (Maris and Oostenveld, 2007) in EEGLAB v. 9 program package (http://sccn.ucsd.edu/eeglab/) involving 2000 permutations. This analysis controls for the Type I error associated with the large number of components and time points. A 95% confidence level was always employed.

## Results

### Behavioral measures

Subjects reported moderate pricking pain during laser stimulation. The mean intensity of laser stimulation was 1.50 ± 0.18 J (mean ± *SD*), corresponding to fluence levels of 11.94 ± 1.45 J/cm^2^. Subjective pain, evaluated after each laser stimulus, was analyzed using one-way ANOVA for repeated measures. Pain was affected by picture type [*F*_(2, 30)_ = 18.4, *P* < 0.001, ε = 0.831]. The mean values ± standard errors of the mean of pain in each emotional condition are listed in Table 1A.

**Table 1:** **Effects of emotional pictures on subjective measures (A), and independent components during LEP period (B) and peri-stimulus period (C)**.

	**Positive picture**	**Neutral picture**	**Negative Picture**	**Negative ÷ Neutral**	**Negative ÷ Positive**	**Neutral ÷ Positive**
**(A) PAIN, VALENCE, AND AROUSAL**						
Pain	2.78 ± 0.10	2.83 ± 0.15	3.34 ± 0.15	*t* = 4.71, *P* < 0.001	*t* = 4.77, *P* < 0.001	*t* = 0.65, *P* = 0.52
Valence	7.35 ± 0.14	5.31 ± 0.13	2.40 ± 0.16	*t* = 16.6, *P* < 0.001	*t* = 19.5, *P* < 0.001	*t* = 12.1, *P* < 0.001
Arousal	4.27 ± 0.38	2.94 ± 0.41	6.07 ± 0.29	*t* = 11.3, *P* < 0.001	*t* = 5.97, *P* < 0.001	*t* = 4.4, *P* < 0.001
**IC_**L**_[ms to ms]**	**Positive picture**	**Neutral Picture**	**Negative picture**	**Negative ÷ Neutral**	**Negative ÷ Positive**	**Neutral ÷ Positive**
**(B) LASER EVOKED POTENTIAL COMPONENTS**						
IC_L_2 [149 to 155]	0.45 ± 0.26	0.24 ± 0.27	0.81 ± 0.27	*t* = 3.1, *P* = 0.008	*t* = 1.87, *P* = 0.08	*t* = 1.11, *P* = 0.29
IC_L_3 [271 to 290]	1.20 ± 0.31	1.12 ± 0.26	1.70 ± 0.26	*t* = 2.39, *P* = 0.03	*t* = 1.98, *P* = 0.07	*t* = 0.69, *P* = 0.50
IC_L_4 [190 to 198]	0.12 ± 0.37	0.22 ± 0.32	0.97 ± 0.34	*t* = 2.50, *P* = 0.024	*t* = 3.51, *P* = 0.003	*t* = 0.28, *P* = 0.78
IC_L_9 [164 to 187]	0.93 ± 0.21	0.35 ± 0.24	0.55 ± 0.24	*t* = 1.36, *P* = 0.19	*t* = 2.92, *P* = 0.01	*t* = 4.43, *P* < 0.001
IC_L_13 [150 to 172]	0.36 ± 0.26	−0.1 ± 0.22	−0.44 ± 0.2	*t* = 1.55, *P* = 0.14	*t* = 4.38, *P* < 0.001	*t* = 2.21, *P* = 0.04
**IC_**B**_ [ms to ms]**	**Positive Picture**	**Neutral picture**	**Negative picture**	**Negative ÷ Neutral**	**Negative ÷ Positive**	**Neutral ÷ Positive**
**(C) PERI-STIMULUS INDEPENDENT COMPONENTS**						
IC_B_7 [−106 to −96]	0.71 ± 0.20	0.19 ± 0.24	0.15 ± 0.20	*t* = 0.19, *P* = 0.815	*t* = 2.50, *P* = 0.024	*t* = 2.58, *P* = 0.021
IC_B_10 [−180 to −156]	0.70 ± 0.09	1.04 ± 0.10	0.58 ± 0.13	*t* = 4.12, *P* = 0.001	*t* = 2.86, *P* = 0.011	*t* = 0.86, *P* = 0.40
IC_B_10 [−55 to −35]	0.83 ± 0.17	0.86 ± 0.14	0.33 ± 0.16	*t* = 3.05, *P* = 0.008	*t* = 2.81, *P* = 0.013	*t* = 0.27, *P* = 0.79
IC_B_10 [47 to 62]	0.88 ± 0.17	0.51 ± 0.17	0.37 ± 0.17	*t* = 0.95, *P* = 0.36	*t* = 3.12, *P* = 0.007	*t* = 1.77, *P* = 0.09
IC_B_13 [−288 to −258]	0.76 ± 0.13	0.36 ± 0.16	0.21 + 0.13	*t* = 1.08, *P* = 0.30	*t* = 3.31, *P* = 0.004	*t* = 2.50, *P* = 0.024
IC_B_13 [0 to 22]	1.00 ± 0.14	0.37 ± 0.22	0.68 ± 0.19	*t* = 1.89, *P* = 0.358	*t* = 1.54, *P* = 0.14	*t* = 2.80, *P* = 0.013
IC_B_15 [−267 to −230]	0.79 ± 0.15	0.77 ± 0.16	0.35 ± 0.14	*t* = 2.31, *P* = 0.035	*t* = 2.91, *P* = 0.011	*t* = 0.13, *P* = 0.90
IC_B_15 [−114 to −72]	0.74 ± 0.16	0.32 ± 0.15	0.23 ± 0.18	*t* = 0.68, *P* = 0.51	*t* = 2.86, *P* = 0.012	*t* = 2.93, *P* = 0.01

(A) Mean values ± SEM, and paired t-tests of pain, valence, and arousal associated with neutral, negative, and positive pictures. Small values of valence correspond to strong unpleasantness. (B) Descriptive statistical values and paired t-tests in four independent components and select intervals showing a statistically significant effect of emotional pictures during LEP period. Source amplitudes are given in Z-values. (C) Descriptive statistical values and paired t-tests in five independent components (Z-values) showing a statistically significant effect of emotional pictures during peri-stimulus period.

The trend component, modeling increases of pain across positive, neutral, and negative categories of pictures, was predominantly linear [*F*_(1, 15)_ = 22.8, *P* = 0.0002].

Paired *t*-tests (Table 1A) showed that laser stimuli were perceived as more painful during viewing negative than neutral or positive pictures. The difference in pain intensity between neutral and positive pictures was not statistically significant (*P* > 0.05).

The three types of pictures were perceived differently in terms of valence [*F*_(2, 30)_ = 300.2, *P* < 0.001, ε = 0.760] and arousal [*F*_(2, 30)_ = 57.1, *P* < 0.001, ε = 0.987]. Negative emotional pictures were perceived as more unpleasant and more arousing than both neutral and positive pictures (Table 1A). Positive pictures were viewed as more pleasant and arousing than neutral pictures. Effects of picture type were best modeled with a linear trend in valence [*F*_(1, 15)_ = 380.9, *P* < 0.0001] and with a quadratic trend in arousal [*F*_(1, 15)_ = 81.0, *P* < 0.0001].

### Sources activations underlying LEPs

**Figure 1:**
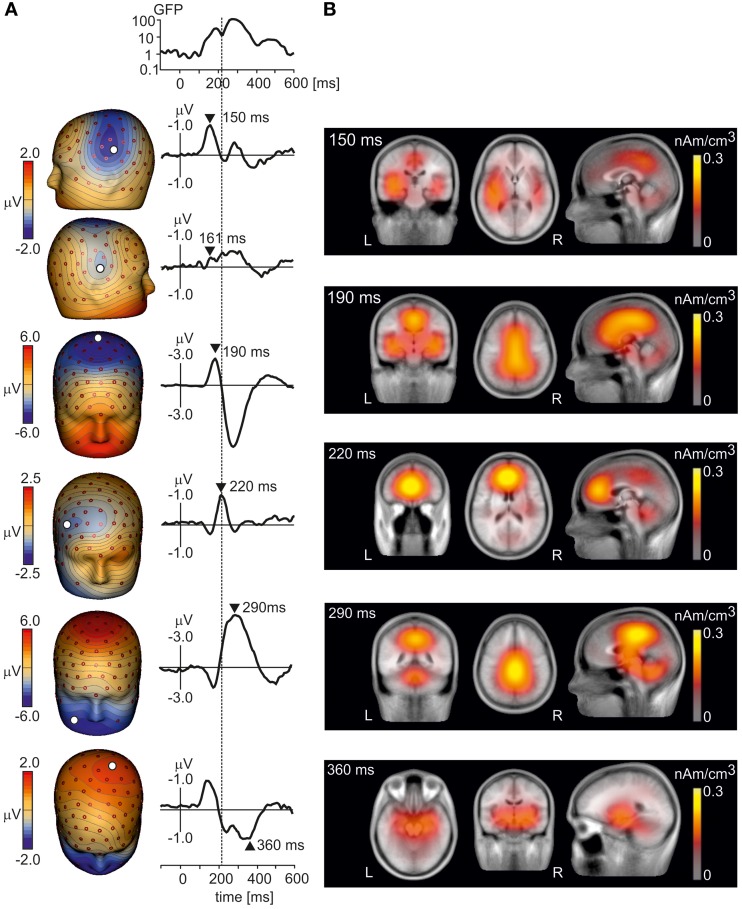
**(A)** The isopotential maps and potentials at select electrodes of grand average LEPs at six time points: 150 ms (N1 potential, top row), 161 ms, 190 ms (N2 potential), 220 ms (N220 potential), 290 ms (N3 potential), and 360 ms (P3 potential, bottom panel). The global field power is also shown. The vertical line crossing all panels indicates the peak latency of the N220 potential. **(B)** Axial (left), transversal (middle) and sagittal (right) views of LAURA activation maps reconstructed from grand average LEP potentials at time points 150 ms (top panel), 190 ms, 220 ms, 290 ms and 360 ms (bottom panel). L, left, R, right.

To illustrate the peak latencies, topographic patterns, and sources of LEP components, LEPs were first analyzed in grand average data in which potential waveforms from all subjects and conditions were averaged. Figure 1A shows the grand average LEP waveforms and isopotential maps of major LEP components. The N1 component was seen in the left and right temporal electrodes during the interval 150–180 ms. The N2 component showed a strong negative maximum at the vertex at 190 ms. The N3 component (Stancak et al., 2013) had a negative maximum at lower face at 290 ms, coinciding with the positive maximum at parietal electrodes also known as P2 component (Garcia-Larrea et al., 2003). However, another positive maximum operating at vertex electrodes and moving later (at 360 ms) to the left parietal region of the scalp was observed, and it is denoted further as P3 component. We also observed a novel LEP component operating at the incisure of the global field power at *t* = 220 ms, which separated the N1/N2 and N3/P2/P3 components. This potential component had a negative maximum at right frontal electrodes and a positive maximum in the left prefrontal region suggesting a source in the medial frontal or prefrontal cortex. The component is denoted as N220 further in the text.

Figure 1B illustrates LAURA source activation maps at select time points corresponding to spatio-temporal maxima of LEP components in Figure 1A. The N1 component was associated with activations in bilateral operculo-insular and temporal regions and in posterior cingulate cortex and precuneus. The N2 component at 190 ms was represented by a strong activation over a large region of the cingulate cortex and bilateral operculo-insular and temporal cortices. The N220 component showed an activation cluster in medial frontal cortex, cerebellum, and posterior cingulate cortex. The N3 component at 290 ms showed the strongest activation clusters in posterior cingulate cortex/precuneus, brainstem, and cerebellum. Finally, activations during the P3 component (360 ms) were seen in bilateral medial temporal cortex. The series of LAURA maps showed a dynamic pattern over the period of LEP with changeable locations of spatial maxima within large activation clusters indicating the presence of multiple components in each cluster.

**Figure 2:**
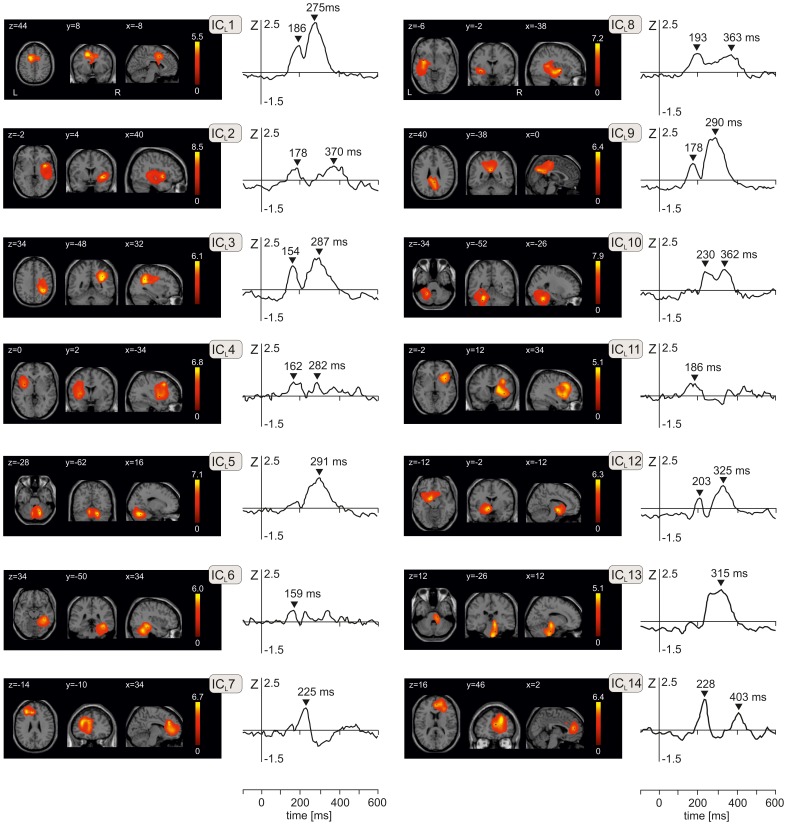
**The component *T*-maps and component time courses of 14 independent components representing activation changes during the period of laser evoked potentials from −100 ms to 600 ms**. Independent components are numbered from IC_L_1 to IC_L_14. In each of 14 panels, transversal, axial, and sagittal views are shown. Values of *x, y*, and *z* indicate coordinates of respective slices in Talairach space. Peak latencies in the component time courses are indicated by black triangles. L, left, R, right.

Figure 2 shows spatial maps and time courses of 14 IC components during LEP period (IC_L_s) obtained from ICA of 700 volumes of source activation in every subject and in each of three emotional stimuli using a group ICA. The Talairach coordinates of components cluster maxima, their anatomical labels and *T*-values are listed in Table 2A. All clusters were statistically significant (*P* < 10^−6^, corrected for multiple tests using family-wise error correction method in SPM8), and therefore a fixed threshold of *T* = 70 was applied throughout. All 14 components explained 92.7% of the total variance.

**Table 2A:** **Anatomical labels, Talairach coordinates, cluster sizes, and *T*-values of independent components clusters seen during LEP period (IC_L_)**.

**IC**_**L**_	**Anatomical label**	***x, y, z***	***k***	***T***
IC_L_1	Left middle frontal gyrus, BA6	−18, 4, 46	28	85.5
IC_L_1	Right anterior mid-cingulate, cortex, BA32	18, 4, 39	–	70.5
IC_L_2	Right anterior insula	39, 4, −4	70	253.7
IC_L_2	Right superior temporal lobe, BA22	39, −39, 4	–	158.3
IC_L_3	Right superior parietal lobule, BA5	32, −46, 25	74	128.3
IC_L_3	Right postcentral gyrus, BA1	39, −18, 39	–	87.8
IC_L_3	Right inferior parietal lobule, BA40	46, −32, 39	–	80.3
IC_L_4	Left middle frontal gyrus, BA8/6	−32, 18, 39	153	148.3
IC_L_4	Left anterior/middle insula	−32, −4, 11	–	112.5
IC_L_4	Left anterior insula	−32, 11, 4	–	110.8
IC_L_5	Right posterior cerebellum, pyramis	18, −67, −25	154	161.5
IC_L_5	Left anterior cerebellum, declive	−11, −67, −18	–	159.7
IC_L_6	Right superior temporal gyrus, BA39/22	32, −53, 25	100	117.8
IC_L_6	Right fusiform gyrus, BA37	39, −39, −11	–	105.6
IC_L_7	Left middle frontal gyrus, BA9	−32, 32, 25	296	201.5
IC_L_7	Left medial frontal gyrus, BA10/8	−11, 32, 32	–	191.3
IC_L_7	Left rostral anterior cingulate cortex, BA32	−11, 45, 4	–	162.4
IC_L_8	Left insula, BA13	−39, −4, −4	214	186.4
IC_L_8	Left parahippocampal gyrus, hippocampus	−32, −11, −11	–	173.5
IC_L_8	Left temporal lobe, BA22	−39, −39, 11	–	151.4
IC_L_9	Right precunues, BA31	4, −67, 32	280	179.6
IC_L_9	Left posterior cingulate cortex, BA23	−4, −45, 25	–	155.9
IC_L_9	Left medial frontal gyrus, BA6	−11, −22, 53	–	152.1
IC_L_10	Left anterior cerebellum	−25, −53, −32	103	156.8
IC_L_10	Left fusiform gyrus, BA37	−39, −46, −18	–	118.8
IC_L_10	Left anterior cerebellum	−32, −39, −25	–	117.3
IC_L_11	Right middle frontal gyrus, BA46	45, 39, 25	182	119.8
IC_L_11	Right middle frontal gyrus, BA9	32, 18, 32	–	117.5
IC_L_12	Left parahippocampal gyrus, BA34	−11, −4, −11	112	144.4
IC_L_12	Left anterior insula	−39, 11, −11	–	96.2
IC_L_13	Right brain stem, mesencephalon	11, −25, −11	38	109.6
IC_L_13	Right brain stem, pons	4, −25, −46	–	107.7
IC_L_14	Right superior frontal gyrus, BA9	18, 46, 32	321	201.9
IC_L_14	Right rostral cingulate cortex (BA32)	18, 32, 4	–	170.4

Activation clusters were thresholded at T = 70. All T-values were statistically significant at P < 10^−6^ (corrected for multiple tests). k, number of contiguous voxels, BA, Brodmann area.

The N1 and N2 potentials (150–200 ms) were represented by IC_L_1 (anterior mid-cingulate cortex premotor cortex, 186 ms), IC_L_2 (right operculo-insular and temporal cortex, 178 ms), IC_L_3 (right superior and inferior parietal lobule and primary somatosensory cortex, 154 ms), IC_L_4 (left anterior insula/frontal operculum and left middle frontal gyrus, 162 ms), IC_L_6 (right fusiform gyrus and temporal cortex), IC_L_8 (left posterior insula/parietal operculum and left medial temporal cortex, 193 ms), IC_L_9 (posterior cingulate cortex/precuneus and left medial frontal gyrus, 178 ms), IC_L_11 (right frontal and prefrontal cortex, 186 ms), and a small peak seen in IC_L_12 at 203 ms (left anterior insula and left parahippocampal gyrus).

The novel N220 potential was represented by IC_L_7 and IC_L_14. IC_L_7 peaked at 225 ms and had clusters in the left rostral anterior cingulate cortex and left prefrontal cortex (Table 2A). IC_L_14 in the right hemisphere was stronger than IC_L_7, and peaked at 228 ms and again at 403 ms. IC_L_14 occupied the right rostral anterior cingulate cortex and the right prefrontal cortex.

The P2/N3 and P3 potentials (230–400 ms) were largely accounted for by IC_L_1 (anterior mid-cingulate cortex and premotor cortex, 275 ms), IC_L_3 (, 287 ms), IC_L_5 (right cerebellum, 291 ms), IC_L_9 (posterior cingulate cortex/precuneus, 290 ms), IC_L_10 (left cerebellum, 230 ms and 362 ms), IC_L_13 (brainstem, 315 ms), and IC_L_12 (left anterior insula and left parahippocampal gyrus). The brainstem component (IC_L_13) had two spatial maxima, one in the mesencephalon and another in pons (Figure 2 and Table 2A).

### Effects of emotional stimuli on LEP independent components

**Figure 3:**
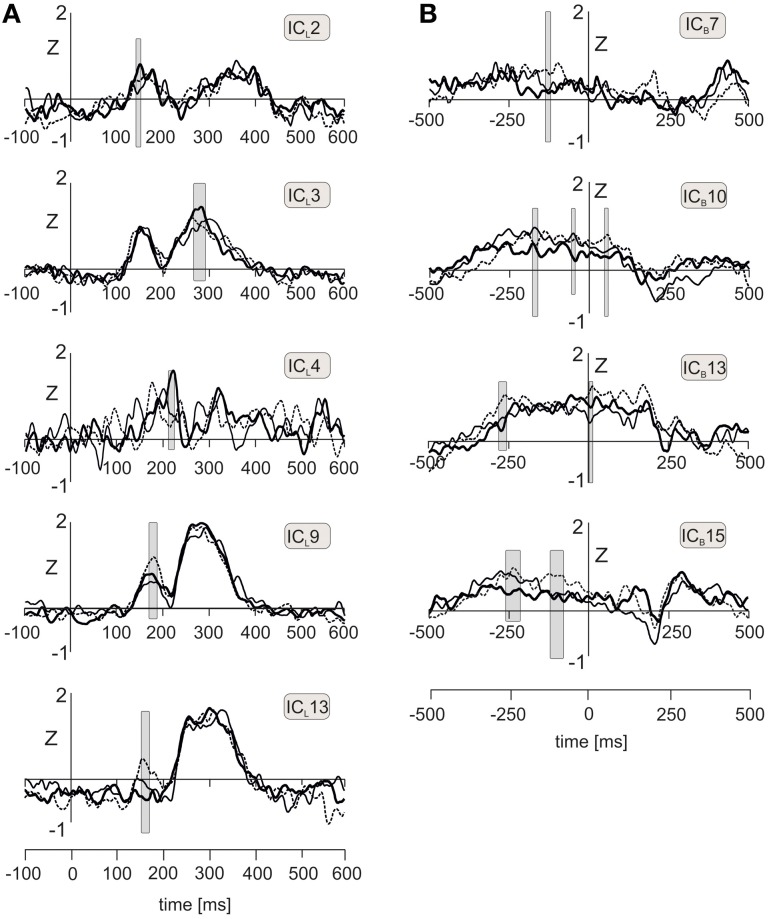
**(A)** Time courses of five independent components of LEPs, labeled IC_L_2, IC_L_3, IC_L_4, IC_L_9, and IC_L_13, during viewing neutral (solid line), positive (dot line), and negative (bold line) emotional pictures. Gray shaded rectangles indicate the time intervals during which one-way ANOVA for repeated measures achieved statistical significance (*P* < 0.05). **(B)** Time courses of four independent components (IC_B_7, IC_B_10, IC_B_13, IC_B_15) showing statistically significant differences between neutral (solid line), positive (dot line), and negative (bold line) emotional pictures during peri-stimulus interval. Gray shaded rectangles indicate intervals showing statistically significant effects of emotional pictures in one-way ANOVA for repeated measures.

The time courses of all 14 IC_L_s in every subject and in each of three emotional conditions were analyzed using one-way ANOVA for repeated measures. Permutation analysis involving 2000 tests in random subsamples was applied to avoid Type 1 error due to large number of ANOVA tests required. Statistical significance was evaluated in the epoch 100–450 ms containing all LEP components. Figure 3A shows the time courses of five IC_L_s showing statistically significant effects of emotional stimuli (*P* < 0.05, corrected for multiple tests). Three components (IC_L_2, IC_L_3, IC_L_4) showed the strongest source activation during negative pictures, and two components (IC_L_9, IC_L_13) showed the strongest activations during positive pictures. Table 1B gives mean values, *F-* and *P*-values representing main effect of pictures in one-way ANOVA for repeated measures, and pair-wise *T*-tests for each of five IC_L_s.

IC_L_2 (right anterior insula and right superior temporal gyrus) in the epoch 149–155 ms showed a statistically significant effect of emotional pictures [*F*_(2, 30)_ = 4.66, *P* = 0.017, ε = 0.997] consisting of a larger activation in negative than neutral emotional condition; the contrast between negative and positive pictures was not statistically significant. The profile of mean values across positive-neutral-negative pictures was best modeled by a quadratic trend [*F*_(1, 15)_ = 5.97, *P* = 0.027]. In IC_L_3 (right anterior and posterior parietal cortex, epoch 271–290 ms), source activity was stronger in negative than neutral pictures [*F*_(2, 30)_ = 4.3, *P* = 0.039, ε = 0.689], and effect of pictures followed a quadratic trend [*F*_(1, 15)_ = 5.23, *P* = 0.037]. In IC_L_4, located in the left anterior and middle insula and left middle frontal gyrus, the source activity in the interval 190–198 ms was stronger in negative than both, neutral and positive pictures [*F*_(2, 30)_ = 4.7, *P* = 0.024, ε = 0.836], and the effect of pictures was linear [*F*_(1, 15)_ = 5.9, *P* = 0.003].

IC_L_9 and IC_L_13 showed increases during viewing positive emotional pictures. In IC_L_9 [posterior cingulate cortex/precuneus, 164–187 ms; *F*_(2, 30)_ = 9.48, *P* = 0.006, ε = 0.984], component activation was stronger in positive than both neutral or negative pictures, which effect followed a quadratic trend [*F*_(1, 15)_ = 10.3, *P* = 0.006]. In IC_L_13 [brainstem, 150–172 ms; *F*_(2, 30)_ = 7.7, *P* = 0.002, ε = 0.689], component activation was the strongest during positive pictures and decreased linearly toward negative pictures [*F*_(1, 15)_ = 19.2, *P* = 0.0005].

None of the laser evoked independent components showed a statistically significant covariation effect with pain ratings in analysis of covariance for repeated measures using three emotional conditions as independent variables, and pain ratings, picture valence and arousal as covariates. However, the strength of IC_L_2 (right anterior insula/frontal operculum and right temporal cortex) covaried with emotional valence of pictures [*F*_(1, 27)_ = 8.41, *P* = 0.007, regression coefficient = −0.53]. The covariation effect of picture arousal was not statistically significant [*F*_(1, 27)_ = 2.47, *P* = 0.13], and since analysis of covariance controls for inter-correlations between covariates, we conclude that only the unpleasantness of pictures contributed to the amplitude increase of IC_L_2 during negative emotional pictures.

### Peri-stimulus activation components

**Figure 4:**
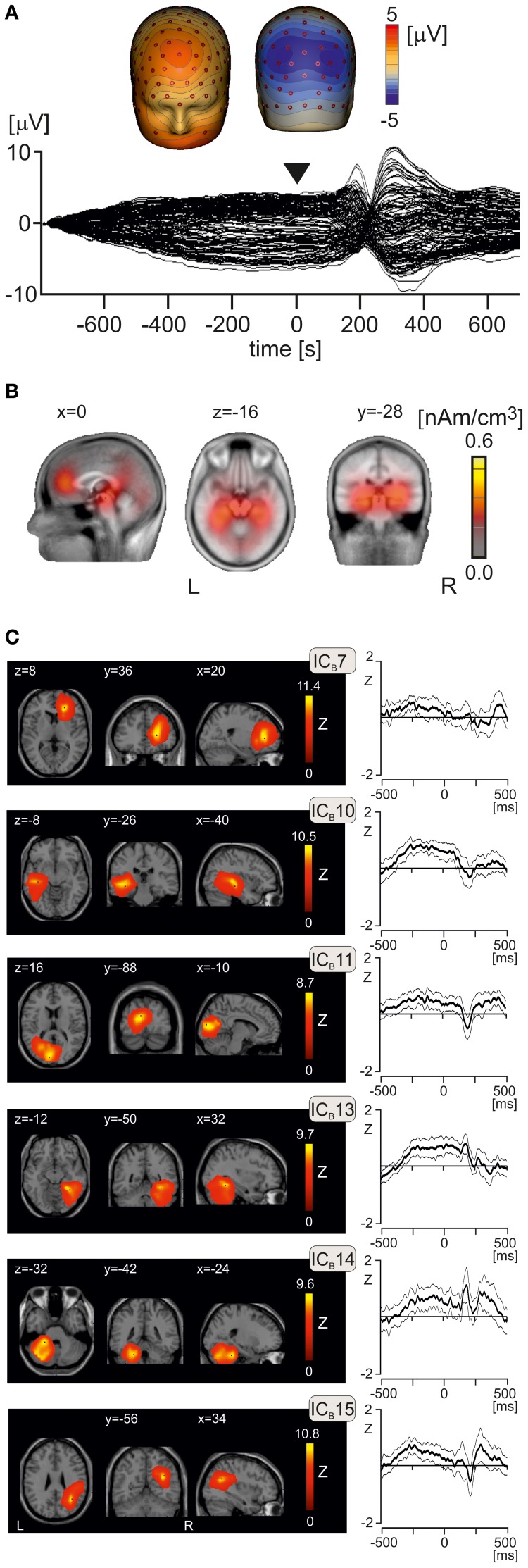
**Peri-stimulus potentials and independent activation components. (A)** The butterfly plot of grand average EEG potential and isopotential maps at time *t* = 0, corresponding to the onset of laser stimulus. The butterfly plot shows comparatively stable potentials during peri-stimulus interval with two symmetric negative spatial maxima at posterior parietal regions, and positive spatial maxima in frontal midline and right frontal electrodes. **(B)** Sagittal, transversal, and axial views of LAURA activation clusters at *t* = 0 s. The Talairach x, y, and z coordinates (mm) of slices are also shown. L, left, R, right. **(C)** Mean component time courses (bold lines) of six independent components (IC_B_7, IC_B_10, IC_B_11, IC_B_13, IC_B_14, IC_B_15) manifesting non-zero amplitude changes in the interval from −100 to 500 ms. Two thin lines paralleling the mean component time courses represent the 95% confidence intervals. L, left, R, right.

To analyse effects of emotional pictures on EEG potentials occurring prior to and at onset of laser stimulus, epochs ranging from −800 to 700 ms were analyzed using combined LAURA and ICA analysis. This long epoch allowed us to analyse whether any activation components showing amplitude increases in peri-stimulus interval (from −500 to +100 ms) during viewing negative pictures would also show similar increases during the LEP period (from +130 to +450 ms). The low cut-off limit was set to 0.1 Hz to allow manifestation of slow potential changes potentially associated with processing of emotional pictures. Figure 4A shows the butterfly plot of grand average EEG potentials, obtained by averaging data from all subjects and conditions. The interval from −500 to +100 ms showed comparatively stable, non-zero potentials with two distinct negative spatial maxima in the left and right posterior parietal electrodes and two positive maxima located in the frontal midline region and lower face (Figure 4A). Figure 4B illustrates cortical activations underlying the topographic map shown in Figure 4A as reconstructed using LAURA. Two dominant activation foci were seen in the left and right medial temporal cortex, and additional weak activations in medial prefrontal cortex, cingulate cortex, and cerebellum.

After inspection of results of ICASSO analysis, which was initially carried out with 20 ICs during peri-stimulus interval (IC_B_s), 17 IC_B_s were computed in group ICA involving all subjects and three emotional conditions. We analyzed the time courses of all 17 IC_B_s to identify the components showing a statistically significant increase of source activation in the peri-stimulus interval from −500 to +100 ms. Figure 4C shows the spatial maps and mean time courses of 6 IC_B_s manifesting statistically significant peri-stimulus changes of source activation evaluated according to a comparatively non-stringent criterion of deviation of the lower 95% confidence limit from zero. Component IC_B_7 represented the right rostral anterior cingulate cortex, caudate nucleus, and right middle frontal gyrus (Table 2B). IC_B_10 referred to activation of the left temporal lobe and left fusiform gyrus. IC_B_11 involved two clusters in the occipital cortex. IC_B_13, similar to IC_B_10 in the left hemisphere, occupied the right temporal lobe and right fusiform gyrus and right cerebellum. IC_B_14 was located in the left cerebellum, and IC_B_15 in the right inferior parietal lobule, precuneus, and middle temporal gyrus (Table 2B).

**Table 2B:** **Anatomical labels, Talairach coordinates, cluster sizes, and *T*-values of independent components clusters seen during peri-stimulus period (IC_B_)**.

**IC**_**B**_	**Anatomical label**	***x, y, z***	***k***	***T***
IC_B_7	Right rostral cingulate cortex, BA32	18, 32, 11	191	123.2
IC_B_7	Right caudate nucleus	18, 18, 4	–	107.1
IC_B_7	Right middle frontal gyrus, BA9	39, 11, 25	–	69.4
IC_B_10	Left superior temporal gyrus, BA41	−39, −32, 4	117	101.9
IC_B_10	Left medial temporal gyrus, BA21/22	−46, −25, −4	–	96.5
IC_B_10	Left fusiform gyrus, BA37	−53, −60, −11	–	55.9
IC_B_11	Left calcarine sulcus, BA17	−11, −81, 11	118	54.9
IC_B_11	Left calcarine sulcus, BA17	−25, −67, 11	78	54.5
IC_B_13	Right temporal lobe, fusiform gyrus, BA37	25, −46, −11	121	94.4
IC_B_13	Right anterior cerebellum, tonsil	25, −46, −32	–	74.9
IC_B_14	Left posterior cerebellum, tuber	−39, −74, −25	99	77.8
IC_B_14	Left anterior cerebellum	−25, −39, −25	–	72.3
IC_B_15	Right middle temporal gyrus, BA39	32, −60, 25	71	110.3
IC_B_15	Right precuneus, BA31/7	18, −60, 32	–	80.1
IC_B_15	Right inferior parietal lobule, BA40/41	46, −32, 32	–	79.3

All T-values, thresholded at T = 50, were statistically significant at P < 10^−6^ (corrected for multiple tests).

The rest of IC_B_s had flat time courses during peri-stimulus interval. These components displayed changes during the LEP period and therefore, are not shown.

Figure 3B shows the component time courses of IC_B_s manifesting both a statistically significant increase of source activation in peri-stimulus period and an effect of emotional pictures. In IC_B_7 [right rostral anterior cingulate cortex and right prefrontal cortex, from −106 to −96 ms; *F*_(2, 30)_ = 4.2, *P* = 0.027, ε = 0.979], positive pictures were associated with a stronger component activation than both neutral and negative pictures (Table 1C). This effect followed a linear trend [*F*_(2, 30)_ = 6.3, *P* = 0.024]. The largest effects of emotional pictures were seen in IC_B_10 (left fusiform gyrus and temporal cortex) in three epochs (from −180 to −156 ms; from −55 to −35 ms; from 47 to 62 ms) all showing the smallest component activation in negative picture condition. In the epochs from −180 to −156 ms [*F*_(2, 30)_ = 7.48, *P* = 0.002, ε = 0.934] and from −55 to −35 ms [*F*_(2, 30)_ = 6.54, *P* = 0.006, ε = 0.928], both neutral and positive pictures showed stronger activation in the left temporal cortex than negative pictures (Table 1C). During the early post-stimulus interval [47–62 ms, *F*_(2, 30)_ = 4.5, *P* = 0.027, ε = 0.837], source activation was the strongest during neutral pictures.

In the IC_B_13 (right fusiform gyrus, temporal cortex and cerebellum, from −288 to −258 ms; *F*_(2, 30)_ = 6.65, *P* = 0.005, ε = 0.964], component activation was the strongest in positive pictures and linearly declined toward negative pictures [*F*_(1, 15)_ = 11.0, *P* = 0.005]. In the interval from 0 to 22 ms, positive pictures also showed the strongest amplitude of IC_B_13 [*F*_(2, 20)_ = 4.96, *P* = 0.017, ε = 1.0]. In IC_B_15 (right inferior parietal lobule, precuneus and temporal cortex), positive pictures showed the strongest and negative pictures the smallest activation in the epoch from −267 to −230 ms [*F*_(2, 30)_ = 4.96, *P* = 0.017, ε = 0.888] as well as in the epoch from −114 to −72 ms [*F*_(2, 30)_ = 6.2, *P* = 0.008, ε = 0.897] (Table 1C).

None of the peri-stimulus intervals showed any statistically significant covariations with pain, picture valence, or arousal (*P* > 0.05).

## Discussion

Our data show augmentation of pain during viewing negative emotional pictures which has been also reported in previous studies (de Wied and Verbaten, 2001; Kenntner-Mabiala and Pauli, 2005; Godinho et al., 2006; Kenntner-Mabiala et al., 2008; de Tommaso et al., 2009; Roy et al., 2009; Ploner et al., 2011). In the peri-stimulus interval, none of the components showed stronger amplitudes during negative than positive or neutral emotional pictures, and in each independent component the positive emotional pictures showed the strongest activation in at least one time interval. In contrast, during the LEP period, three independent components showed stronger activations during negative emotional pictures. Activation in the right anterior insula/temporal cortex correlated with emotional valence of pictures. Although negative and positive pictures differed in both valence and arousal ratings, we have controlled for individual contributions of both these variables, as well as pain, using one-way analysis of covariance for repeated measures.

### LEP activation components

Thanks to implementation of LAURA and decomposition of source activation map series into independent components, we were able to demonstrate activations in many regions of the brain of which some e.g., in bilateral operculo-insular cortex (Tarkka and Treede, 1993; Bromm and Chen, 1995; Garcia-Larrea et al., 2003; Kakigi et al., 2005), anterior mid-cingulate (Garcia-Larrea et al., 2003; Kakigi et al., 2005; Stancák et al., 2010), posterior cingulate cortex/precuneus (Bromm, 2004; Barrett, 2009; Stancak et al., 2013), and parahippocampal cortex (Valeriani et al., 1996, 2000, 2002; Watanabe et al., 1998; Stancak et al., 2013) have been reported in previous laser evoked potential studies and others are shown for the first time.

One of the novel LEP components was the negative potential peaking at 220 ms at the right frontal region of the scalp (Figure 1A) which purportedly originated in the right and, to a lesser extent, in the left rostral anterior cingulate cortex and ventromedial prefrontal cortex (IC_L_14 and IC_L_7). This component was strongest when the rest of the components returned to baseline, or had not yet begun. In addition, the right rostral anterior cingulate/and ventromedial prefrontal cortex was also active during the peri-stimulus interval in the form of IC_L_7. The rostral anterior cingulate cortex is a prominent part of the resting default mode network (Raichle et al., 2001; Raichle and Snyder, 2007; Laird et al., 2009). Therefore, we conjecture that the N220 component may be a manifestation of a brief activation of a resting state network during the comparatively silent period separating the early, purportedly sensory (<200 ms), and late (>230 ms), higher-order stage of nociceptive processing. The N220 component of LEP was identified in the context of visual emotional stimuli and therefore, it may have also resulted from interaction of both types of stimuli. Further validation of this novel component based on LEP data obtained in absence of concurrent visual stimulation is required.

Other novel components of LEPs, located in brainstem (IC_L_13) and cerebellum (IC_L_5 and IC_L_10), also necessitate further confirmation which could be accomplished, for instance, by acquiring combined EEG-fMRI recordings. However, the presence of brainstem activation after application of a single laser stimuli is consistent with fMRI studies reporting activation of multiple brainstem regions such as rostral ventro-medial medulla and peri-aqueductal gray matter during noxious heat stimulation (Ghazni et al., 2010; Cahill and Stroman, 2011), and with a recent evoked potential study demonstrating a brainstem dipole during noxious esophageal stimulation (Oosterwijk et al., 2012). Brainstem houses several important regions of the descending nociceptive control system including rostral ventromedial medulla, peri-aqueductal gray matter, and dorsolateral pontine tegmentum (Fields, 2004). Therefore, we conjecture that the brainstem component may represent activations in regions belonging to the descending nociceptive control system.

Nociceptive fibers project to the anterior cerebellum via climbing fibers receiving from the spino-olivocerebellar tract neurons located in ipsilateral dorsal funiculus of the spinal cord (Barrett, 2006). Noxious skin stimulation in cats produces large field potentials recordable on the cerebellar surface (Lindquist et al., 2012). However, the cerebellar components in our study were active predominantly during the late N2 and N3 periods suggesting their role in the higher-order, post-sensory processing. In spite of a large number of functional brain imaging studies reporting cerebellar activations during noxious stimulation, evidenced by representation of cerebellar activations in two large meta-analysis studies (Farrell et al., 2005; Duerden and Albanese, 2013), the role of cerebellum in nociceptive processing remains unclear (Saab and Willis, 2003; Barrett and Bliss-Moreau, 2009). A recent study showed that cerebellum is a part of a network, along with anterior insula and hippocampal formation, mediating emotional modulation of pain (Moulton et al., 2011), emphasizing the role of cerebellum in higher-order, cognitive and emotional control of pain. Cerebellar potentials in human EEG recordings are obscured by caudal location of cerebellum and comparatively thick layer of muscle tissue in posterior neck region. To our knowledge, electrophysiological responses to noxious stimulation in human cerebellum have not been reported previously and therefore, this finding is preliminary and requires further validation.

The present study has not identified an activation cluster in the contralateral primary somatosensory cortex (SI). Such sources have been demonstrated in a few previous LEP studies (Tarkka and Treede, 1993; Valeriani et al., 1996; Inui et al., 2003; Schlereth et al., 2003; Baumgärtner et al., 2011; Stancak et al., 2013). As the volume of the primary somatosensory cortex activated with focal laser stimulus is small, we assume that the combination of a comparatively large voxel size and smoothness of LAURA employed in the present study might have contributed to the lack of distinct activation in the contralateral SI.

### Emotional modulation of LEP independent components

Three of the LEP independent components, located in the right anterior insula and temporal cortex, left anterior and middle insula, and right superior and inferior parietal lobule (IPL) and SI, showed the largest amplitude during negative emotional pictures. Involvement of anterior insula in emotional modulation of pain has been reported in recent fMRI studies (Roy et al., 2009; Moulton et al., 2011; Ploner et al., 2011) and one LEP study (Stancak et al., 2013). Anterior insula is also frequently reported among activation clusters in studies of emotions (Phan et al., 2002). The right anterior insular/temporal cortex showed a correlation with emotional valence of pictures, indicating that these structures are engaged in monitoring negative emotional stimuli occurring in parallel with painful stimuli. Anterior insula is activated during viewing facial expressions of disgust (Wicker et al., 2003), smelling unpleasant odors (Heining et al., 2003; Wicker et al., 2003), and loss aversion during decision making (Knutson et al., 2007). Although emotion-induced activation changes were not related to pain rating variations in the present study, data suggest that negative emotions affect nociceptive processing, particularly in the right anterior insula. Our finding of valence modulation of the right anterior insula activation fits with the proposed role of anterior insula in cross-modal integration of senses (Calvert, 2001) and in creation of “global emotional moments” in awareness (Craig, 2009).

The right IPL has been consistently reported in brain imaging studies involving noxious stimulation; in a recent meta-analysis study of pain-related activation clusters (Duerden and Albanese, 2013), the right IPL was represented by three clusters of which the strongest cluster (*x* = 46 mm, *y* = −38 mm, *z* = 42 mm) closely corresponded to the cluster maximum seen in IC_L_3 (*x* = 46 mm, *y* = −32 mm, *z* = 39 mm). IPL subserves many functions such as integration of visual and somatosensory information and “wanting to move” type of movement intention (Desmurget and Sirigu, 2012). It can be speculated that activation of IPL may prime the executive networks prompting a protective limb movement by increasing the “wanting to move” intention. The finding of accelerated motor reactions during viewing negative emotional pictures would accord with this explanation (Coombes et al., 2007). Other regions involved in IC_L_3 and showing amplitude increases during negative emotions were the superior parietal lobule (Brodmann area 5), and primary somatosensory cortex (Brodmann area 1). Thus, it appears that negative emotional pictures boost nociceptive activation in a large portion of the right primary and higher-order somatosensory cortex.

Two components, IC_L_13 (brainstem) and IC_L_9 (posterior cingulate cortex/precuneus) showed strongest activation during processing of noxious stimulus in the context of positive emotional stimulus. In IC_L_13, a comparatively strong activation peak was only seen during viewing positive emotional pictures in the early latency epoch (164–187 ms), and the activation was weakest during negative emotional stimulus. It can be speculated that such early activation of brainstem in the presence of a positive emotional stimulus may be associated with a more efficient functioning of the descending nociceptive controls during positive than negative emotional stimuli, which may contribute to the greater pain seen in negative than positive or neutral emotional pictures. In support of this explanation, the strength of the diffuse noxious inhibitory control system, operating via the brainstem endogenous opioid system, has been shown to be changed by cognitively altered pain intensity (Nir et al., 2012). As far as the posterior cingulate cortex and precuneus is concerned (150–172 ms), these default mode network regions have also been shown to maintain activation during visual, auditory (Greicius and Menon, 2004) and noxious electro-cutaneous stimulation (Mantini et al., 2009). Therefore, it is not surprising that this activation cluster has also been found during noxious laser stimulation. Interestingly, one recent study showed an increased functional connectivity between brainstem and posterior cingulate cortex/precuneus during acupuncture treatment (Zyloney et al., 2010). Such functional coupling between both regions and similar amplitude increases during positive emotional pictures in overlapping epochs suggest augmentation of one functional network during noxious laser stimulation, encompassing the posterior cingulate cortex/precuneus and brainstem.

Interestingly, none of the components showed a statistically significant covariation with pain ratings. Lack of correlation between IC_L_s amplitudes and pain ratings suggests that augmentation of pain during viewing negative emotional picture may have been formed later, e.g., during the period of reporting numeric ratings by repeated button presses, which process may reflect a variety of factors such as emotional valence of pictures, ability to recall pain sensation, and motor readiness. However, the lack of statistically significant covariation effects between pain ratings and brain activations may be also attributed to the comparatively low sensitivity of the numerical pain rating scale which was used to report pain on each trial. Most of the subjects gave pain values ranging from 2 to 4 or from 3 to 5. Such a narrow range of pain levels was sufficient to detect the statistically significant effects of emotional pictures in averaged data, however, it may have not been sensitive enough for mapping pain rating values onto brain activation. Further, we avoided the use of pictures involving mutilated bodies or erotic scenes in our set of pictures. These two types of picture produce the strongest cortical potentials (Briggs and Martin, 2009; Weinberg and Hajcak, 2010) and therefore, would offer larger variations between and within positive and negative type of pictures which might potentially might favor covariations between pain ratings and cortical responses.

### Peri-stimulus interval

Our study analyzed for the first time the visually-induced, emotion-related brain activations during the interval just preceding a laser stimulus, which interval is typically used to compute and subtract baseline levels in evoked potential studies. We demonstrated that the cortical activation prior to a laser stimulus shows a stable, non-zero topographic potential field. This peri-stimulus activation is maintained by a set of cerebral regions involving visual cortex, bilateral temporal and fusiform cortices, rostral anterior and prefrontal cortex, right inferior parietal lobule, and cerebellum. Some of the activations, such as those in bilateral fusiform gyri/temporal cortices were sensitive to emotional type of the picture. However, none of the activation components showed the strongest activation during negative emotional stimulation or any covariation effect with pain intensity or emotional valence ratings. Thus, present data found little support for the background activation model as explanation of augmented pain during negative emotional picture as such model would only be compatible with stronger brain activations during negative emotional pictures and a match between component activations and pain and/or valence ratings across three emotional conditions. Our data suggest that the peri-stimulus background activation possibly has only a permissive effect on emotional modulation of pain consisting in decreasing the resting level of activation during negative emotional stimuli, which may aid in rapid recruitment of brain regions participating in pain processing.

The laser stimulus has been applied 1100 ms after onset of a visual stimulus, which latency corresponds to the sustained and stable principal ERP component known as the late positive potential (Foti et al., 2009; Weinberg and Hajcak, 2010), operating in the interval from 600 to 2000 ms. The late positive potential has negative maximum in the parieto-occipital and positive maximum in frontal and central electrodes (Foti et al., 2009), which map was similar to the topographic map shown in Figure 4A in our study. The late positive potential in the previous study showed the strongest activation during neutral pictures and a smaller, comparable activity in positive and negative pictures (Foti et al., 2009). This pattern of activity would be consistent with preponderance of activation in neutral condition in the component IC_B_10 (−250 ms to −150 ms), occupying the left temporal lobe and fusiform gyrus, and IC_B_15 (−250 ms to −150 ms), involving right middle temporal gyrus, precuneus and right inferior parietal lobule. Therefore, we attribute the activations seen in the peri-stimulus interval, ranging (from −500 to +100 ms relative to onset of laser stimulus) to the late positive potential component seen during emotional visual stimulation (Foti et al., 2009; Weinberg and Hajcak, 2010).

Further analysis of peri-stimulus data suggests prevalence of activation in positive emotional pictures in the left and right fusiform gyri and right rostral anterior cingulate cortex prior to and/or shortly after onset of laser stimulus. The infero-temporal cortex activations occupied the anterior regions of bilateral fusiform cortex overlapping or adjoining the fusiform areas known to be activated during viewing emotional faces (Kanwisher et al., 1997) and emotional scenes (Sabatinelli et al., 2011). Activation of temporal and infero-temporal cortex perhaps reflects an ongoing monitoring and evaluation of a broader stimulation context which may be relevant for formation of an adequate behavioral response to pain. Presence of activation in the occipital cortex (IC_B_11) is consistent with ongoing visual activity during picture viewing.

Several independent components seen in the peri-stimulus interval bore a close association to the default mode resting network previously demonstrated in functional brain imaging studies (Raichle et al., 2001; Greicius et al., 2003; Raichle and Snyder, 2007). Laird et al. (2009) conducted a meta-analysis of 62 functional imaging studies regions participating in the default network. Activations seen during peri-stimulus interval in precuneus, rostral anterior cingulate cortex, right middle temporal gyrus, and right inferior parietal lobule closely corresponded to those representing the major default mode network regions, listed in Table 1 in Laird et al. (2009). As these components were stronger during positive or neutral compared to negative emotional stimuli, it is likely that negative emotional pictures have attenuated parts of the default mode network. This explanation is consistent with findings of attenuated activity in parts of default network during induced sad mood (Harrison et al., 2008) or following viewing a fearful video clip (Eryilmaz et al., 2011), and increased default mode activity during viewing strongly pleasing aesthetic pictures (Vessel et al., 2012). It should be pointed out that the discovery of default mode network and its subsequent explorations have been based on hemodynamic measures of brain activations typically occurring on the scales of tens of seconds. There are no data, to our knowledge, documenting transient activation changes of the default mode network regions occurring on the scale of milliseconds. Future experimental studies involving pre-stimulus epochs devoid of concurrent visual stimulation should address the question of resting state network effects on LEPs.

The conceptual act theory of emotions (Barrett, 2006, 2009; Barrett and Bliss-Moreau, 2009) offers additional interpretation framework to our findings. The comparatively stable activations seen during viewing emotional pictures in peri-stimulus interval appear to reflect the core affect, a distributed network of brain regions underlying conscious experience (Barrett and Bliss-Moreau, 2009). Increases of pain seen during negative emotional pictures may refer to correspondence between valence of core affect, shifted by emotional pictures, and pain conceptualization initiated by laser stimulus and incorporating previous pain experience. As the process of conceptualization, eventually ending in categorization of a sensation as pain, occurs “in the blink of an eye” (Barrett, 2006), our data offer a unique insight into dynamic integration of nociceptive (sensory) and background emotional activations. In particular, increased activation of bilateral anterior insular and anterior temporal cortex during viewing negative emotional pictures allude to involvement of the lateral paralimbic group, one of six functional networks operating in emotions (Kober et al., 2008). Other activations in peri-genual and posterior cingulate cortex/precuneus correspond closely to the default mode network which has been also related to the process of conceptualization in emotional situations (Oosterwijk et al., 2012) and represents a stable component of brain activation at rest (Yeo et al., 2011). Indeed, EEG data do not offer yet the possibility to detect all important activation clusters seen in fMRI or PET studies of emotions (Kober et al., 2008; Lindquist et al., 2012), such as hypothalamus, thalamus, or amygdala and therefore, our study might have missed some brain regions adding to the integration of emotion and pain.

We conclude that laser evoked potentials, occurring in context of emotional pictures, are preceded by a comparatively stable activation of bilateral fusiform gyri and adjacent temporal cortex, cerebellum and right rostral anterior cingulate and prefrontal cortex. Prevailing peri-stimulus activation components show the largest activations during positive emotional pictures and therefore, are not likely to account for augmented pain during viewing negative emotional pictures. Laser stimuli activate a large number of brain regions. The left and right operculo-insular and temporal cortex and the right anterior and posterior parietal cortex show activation increases during processing noxious stimuli in the context of negative emotional stimulation. Data suggests that negative emotional stimuli augment activations in select regions of the cerebral pain network not sooner than during the period of nociceptive processing, whilst effects of the background brain activation on emotional modulation of pain appears to be only permissive in the sense of a reduced build up of resting activation in select brain regions during viewing negative emotional scenes.

Our study, although limited by the types of stimuli used, calls for a revision of the methods of event-related potential analysis in studies involving application of a sensory stimulus on the background of an ongoing, parallel task. A standard analysis of event-related potentials involves a baseline correction using a pre-stimulus epoch data, which permits the analysis of peak amplitudes and latencies of event-related potentials. Although baseline correction will set potentials at every electrode to zero during the time of application of a sensory stimulus, the pre-stimulus activation will reappear during the period of an evoked potential despite baseline correction. Thus, event-related potentials represent a complex mixture of pre-stimulus activation components, and those evoked by a sensory stimulus. Implementation of ICA and a detailed analysis of pre-stimulus epochs along with components of event-related potentials appears to be a promising avenue for future event-related potential research.

### Conflict of interest statement

The authors declare that the research was conducted in the absence of any commercial or financial relationships that could be construed as a potential conflict of interest.
